# Editorial: Young People's Sexual and Reproductive Health in Sub-Saharan Africa (SSA): Bridging the Research-to-Practice Gap

**DOI:** 10.3389/frph.2021.820142

**Published:** 2021-12-22

**Authors:** Juliet Iwelunmor, Morenike Oluwatoyin Folayan, Ucheoma Nwaozuru, Oliver Ezechi

**Affiliations:** ^1^Behavioral Science and Health Education, Saint Louis University, St. Louis, MO, United States; ^2^Department of Child Dental Health, Institute of Public Health, Obafemi Awolowo University, Ile-Ife, Nigeria; ^3^Department of Implementation Science, Wake Forest School of Medicine, Winston-Salem, NC, United States; ^4^Department of Clinical Sciences, Nigerian Institute of Medical Research, Yaba, Nigeria

**Keywords:** sexual and reproductive health, implementation science, facilitators and barriers, young people, sub-Saharan Africa

## Introduction

Sexual and reproductive health (SRH) among young people, aged 10–24 years, in Sub-Saharan Africa (SSA) remains a major public health challenge, with evident gaps in access to SRH services and increased risk of poor SRH (1). For example, less than 20% of young people in SSA are aware of their HIV status, despite the region accounting for almost 90% of the world's HIV cases among adolescents and youth (2). Point-of-care tests exist and have the potential to revolutionize the prevention and care of HIV and other STIs, thus interrupting transmission and preventing the sequelae of untreated infections (3, 4). However, the awareness and uptake of such SRH preventive services remain sub-optimal among young people (5). While this research-to-practice gap is widely known, there is limited discussion on how it can be bridged. The collection in this Frontier Research Topic begins to partly remedy the problem by recounting collective efforts to promote young people's SRH in SSA. Here we share our learnings with the hope of advancing the discussion on how to bridge the research-to-practice gap. We summarize the articles in this special issue in three main (and overlapping themes).

## Risk Factors Associated With Youth Sexual and Reproductive Health

Temin et al. open this collection by exploring HIV-related risk determinants from the perspective of foreign migrant adolescent girls and young women in South Africa who participated in an evidence-based female-focused intervention–“*Girls Club Project*.” They provide recommendations for more inclusive HIV prevention programming that reaches foreign migrant adolescent girls and young women. With a similar focus on risk factors, Envuladu et al. assessed the covariates of sexual activity and consistent condom use among adolescents in Plateau State, Nigeria. Their results suggest a need for gender-tailored interventions. Taking a broader perspective, tailored interventions that are contextually appropriate can enhance the acceptability and adoption of SRH interventions.

## Knowledge and Uptake of Sexual and Reproductive Health Services

Two authors highlight knowledge and uptake of Youth Sexual and Reproductive Health services with helpful insights. Yuya et al. assessed the level and predictors of knowledge of reproductive rights among Haramaya University students in Ethiopia. The study revealed that lack of awareness/information on reproductive health issues and absence of reproductive health services utilization were important predictors of participants' knowledge of reproductive rights. This warrants further investigations on whether knowledge of reproductive rights can promote uptake of sexual and reproductive health services. Lawrence et al. shared the work undertaken by the team as part of the Public Health Adolescent Services Evaluation, which is a national evaluation of adolescent HIV services in Kenya. The evaluation revealed a preference for adolescent autonomy in seeking sexual and reproductive health services and contacting healthcare workers for sexual and reproductive health information. This study confirms the value of adolescent and youth-friendly health services and the need for contextually appropriate strategies to enhance uptake and adoption.

## Implementation Science and Young People's Sexual and Reproductive Health

Echoing the call for *Bridging the Research-to-Practice* gap, three articles highlight the application of implementation science in young people's sexual and reproductive health. Mgoli Mwale et al. evaluate the sustainability of the Community Score Card to improve Adolescent Sexual and Reproductive Health in Ntcheu, Malawi. Their work provides compelling evidence of the importance of agents and context in the sustainability of interventions. They specify real-world facilitators, barriers, and opportunities in promoting sustainability within resource-constrained settings. Libous et al. offer a new generalizable approach to guide intervention adaptation that builds on existing infrastructure, culture, and resources to inform implementation strategies. Their work focused on the adaptation of a *trauma-informed cognitive behavioral therapy* intervention addressing mental and sexual health for adolescents and young adults living with HIV guided by the ADAPT-ITT framework. Obiezu-Umeh et al. presented compelling findings from a systematic review of 23 articles on the implementation outcomes and implementation strategies of youth-friendly sexual and reproductive health services in sub-Saharan Africa. The authors underlined the need to develop rigorous studies further to understand better which and what combination of implementation strategies would lead to more gain in promoting the uptake and sustainment of youth-friendly sexual and reproductive health services. These articles provide concrete suggestions for community and young people's engagement in intervention development, adaptation, and implementation processes, to foster co-creation and ownership within communities. These are critical for the adoption, scale-up, and sustainability of interventions as we seek to bridge the research-to-practice gap.

## Conclusion

Individually, these contributions address an array of critical issues. Collectively, they provide momentum on the importance of contextually appropriate, youth-friendly, and youth-engaged approaches to enhance the uptake, scale-up, and sustainability of young people's SRH interventions in SSA. We acknowledge that articles presented in this collection are not exhaustive of the work in the field. Nevertheless, the findings, insights, and perspectives discussed in each paper could inspire new research and interventions.

This special issue continues to highlight substantial structural, social, and individual barriers that limit the uptake of SRH services. Nonetheless, a few articles highlight the importance of active community engagement and active youth participation to circumvent these barriers. Community engagement and youth participation can enhance the uptake, appropriateness, and sustainability of SRH intervention and can help move us closer to the goal of bridging the research-to-practice gap in promoting SRH among young people in SSA. Participatory approaches such as crowdsourcing that involve community members in solving a problem and then publicly sharing innovative solutions could provide an opportunity to co-create with young people and community members (6, 7). This can improve the appropriateness and acceptability of SRH interventions for young people in SSA and, ultimately, sustainability. We, therefore, call for more community-engaged and participatory approaches in the area of SRH for young people in SSA. In closing, we hope this collection will become a reference for continuing research in bridging the knowledge-to-do gap in sexual and reproductive health among young people in SSA.

## Author Contributions

All authors listed have made a substantial, direct, and intellectual contribution to the work and approved it for publication.

## Conflict of Interest

The authors declare that the research was conducted in the absence of any commercial or financial relationships that could be construed as a potential conflict of interest.

## Publisher's Note

All claims expressed in this article are solely those of the authors and do not necessarily represent those of their affiliated organizations, or those of the publisher, the editors and the reviewers. Any product that may be evaluated in this article, or claim that may be made by its manufacturer, is not guaranteed or endorsed by the publisher.
